# The use of metabolomics to dissect plant responses to abiotic stresses

**DOI:** 10.1007/s00018-012-1091-5

**Published:** 2012-08-12

**Authors:** Toshihiro Obata, Alisdair R. Fernie

**Affiliations:** Max Planck Institute of Molecular Plant Physiology, Am Mühlenberg 1, 14476 Potsdam-Golm, Germany

**Keywords:** Metabolomics, Plants, Abiotic stress, Metabolic response, Branched chain amino acid, Enzyme complex

## Abstract

Plant metabolism is perturbed by various abiotic stresses. As such the metabolic network of plants must be reconfigured under stress conditions in order to allow both the maintenance of metabolic homeostasis and the production of compounds that ameliorate the stress. The recent development and adoption of metabolomics and systems biology approaches enable us not only to gain a comprehensive overview, but also a detailed analysis of crucial components of the plant metabolic response to abiotic stresses. In this review we introduce the analytical methods used for plant metabolomics and describe their use in studies related to the metabolic response to water, temperature, light, nutrient limitation, ion and oxidative stresses. Both similarity and specificity of the metabolic responses against diverse abiotic stress are evaluated using data available in the literature. Classically discussed stress compounds such as proline, γ-amino butyrate and polyamines are reviewed, and the widespread importance of branched chain amino acid metabolism under stress condition is discussed. Finally, where possible, mechanistic insights into metabolic regulatory processes are discussed.

**Electronic supplementary material:**

The online version of this article (doi:10.1007/s00018-012-1091-5) contains supplementary material, which is available to authorized users.

## Introduction

When plants face unfavourable growth conditions, abiotic stress retards plant growth and productivity. Under most abiotic stress conditions, plant metabolism is perturbed either because of inhibition of metabolic enzymes, shortage of substrate, excess demand for specific compounds or a combination of these factors and many other reasons. Therefore, the metabolic network must be reconfigured to maintain essential metabolism and to acclimate by adopting a new steady state in light of the prevailing stress conditions. This metabolic reprogramming is also necessary to meet the demand for anti-stress agents including compatible solutes, antioxidants and stress-responsive proteins. The accumulation of reactive oxygen species (ROS) is another problem causing oxidation and dysfunction of cellular components and in the worst case cell death. The optimisation of metabolic flux via the organellar electron transport chains is, moreover, crucial in order to dampen ROS production. Maintenance of the redox state in the cell is thus another important task to provide the reducing power required for ROS scavenging. Despite such important roles of metabolic regulation under stress conditions, our current understanding of this process is fragmented and far from complete.

Metabolomics is a powerful tool by which to gain a comprehensive perspective of how metabolic networks are regulated and has indeed been applied by many researches in recent years. It can, additionally, be used to elucidate the functions of genes as a tool in functional genomics and systems biology approaches. The term “metabolomics” is defined as comprehensive and quantitative analysis of all small molecules in a biological system [1]. The plant kingdom may contain between 200,000 and 1,000,000 metabolites, while for a single species the number may approach a few thousand (the estimate for *Arabidopsis* is ca. 5,000) [2–5]. Indeed the KNApSAck database (http://kanaya.naist.jp/KNApSAcK/) contains around 50,000 metabolite entries in plants described so far in the literature [6]. Due to the large variety of chemical structures and properties of small molecules, there is so far no single technique to identify and quantify all of them. Even the most comprehensive methods detect only between 1,000 and 2,000 molecular features [7, 8]. Several techniques including gas chromatography-mass spectrometry (GC-MS), liquid chromatography (LC)-MS, capillary electrophoresis (CE)-MS and nuclear magnetic resonance spectroscopy (NMR) are commonly used in plant metabolomics research. They are used sometimes in combination since they are largely complimentary with each independent method having preferential coverage of diverse types of metabolite. Here we briefly introduce the advantages and limitations of each method. Thereafter studies employing the metabolomic approach to dissect plant response to abiotic stresses will be discussed. Finally, using data already collected, we attempt to elucidate the general and stress-specific responses.

## Techniques used for plant metabolomic research

### Gas chromatography-mass spectrometry (GC-MS)

Gas chromatography-mass spectrometry is the most widely used technique for plant metabolomics research to date. Polar metabolites are derivatised to render them volatile and then separated by GC. Electron impact (EI) allows robust interfacing of GC with MS resulting in highly reproducible fragmentation patterns. For detection, time-of-flight (TOF)-MS has become the method of choice because of advantages including fast scan times, which give rise to either improved deconvolution or reduced run times for complex mixtures, and high mass accuracy. The crucial advantage of this technology is that it has long been used for metabolite profiling, and thus there are stable protocols for machine setup and maintenance, and chromatogram evaluation and interpretation [9–11]. The robustness of the protocol means that libraries of retention time and mass spectra data for standard compounds can be shared among laboratories [12]. There are several metabolite databases available including the NIST [13], FiehnLib [14] and Golm metabolic databases (GMD, [15]), which are useful tools for peak annotation. Additionally, the short running time and relatively low running cost are also strong advantages of GC-MS. However the use of GC-MS is limited for thermally stable volatile compounds, making the analysis of high molecular weight compounds (larger than 1 kDa) difficult. Due to these characteristics, GC-MS facilitates the identification and robust quantification of a few hundred metabolites in plant samples including sugars, sugar alcohols, amino acids, organic acids and polyamines, resulting in fairly comprehensive coverage of the central pathways of primary metabolism.

### Liquid chromatography (LC)-MS

While GC has a limitation due to volatilisation of compounds, LC does not require prior sample treatment and separates the components in a liquid phase. The choice of columns, including reversed phase, ion exchange and hydrophobic interaction columns, provides the separation of metabolites based on different chemical properties. Therefore, LC has the potential to analyse a wide variety of metabolites in plants. The recent development of ultra-performance liquid chromatography (UPLC) makes the technique more powerful because of its higher resolution, sensitivity and throughput than conventional high-performance liquid chromatography (HPLC) [16]. Electrospray ionisation (ESI) is widely used for ionisation to connect LC and MS. Many types of MS including quadrupole (Q), TOF, qTOF, triple quadrupole (QqQ), ion trap (IT), linear trap quadrupole (LTQ)-Orbitrap and Fourier transform ion cyclotron resonance (FT-ICR)-MS are used depending on the sensitivity, mass-resolution and dynamic range required (see [17, 18] for the detail). The combination of these techniques allows us to identify and quantify a large variety of metabolites even if they have high molecular mass, great polarity and low thermostability. On the other hand the flexibility of the method also causes difficulty in establishing large mass spectral libraries for peak identification because of the instrument-type dependent retention time and mass spectra [19], and forces each research group to create its own “in-house” LC-MS reference library. That said, there are a number of websites that aid in mass-spectral analyses [20], and recent recommendations for metabolite reporting [7] should improve the transparency of the methods used by researchers. Furthermore, isotope labelling as a means of confirming the identity of peaks has recently been proposed and demonstrated to allow the identification of circa 1,000 metabolites using the FT-ICR-MS approach [8, 21]. To date, LC-MS is mainly used with a reverse phase column to analyse secondary metabolites because of its ability to separate compounds with similar structure and to detect a wide range of metabolites. However, it is worth noting that specialised protocols for determining phosphorylated intermediates, which are not readily detected by GC-MS, have also begun to be developed using this technology [22], as have methods for the comprehensive analysis of phytohormones [23].

### Capillary electrophoresis (CE)-MS

Capillary electrophoresis separates polar and charged compounds on the basis of their charge-to-mass ratio. CE is able to separate a diverse range of chemical compounds and is a more powerful technique than LC with respect to separation efficiency [24, 25]. ESI is commonly used for ionisation as in LC-MS, with TOF-MS being the most commonly used detector in CE-MS-based metabolomics studies. This combination provides high mass accuracy and high resolution. The high scan speed of TOF-MS makes this instrument very suitable for full scan analyses in metabolomics. One of the unique properties of CE-MS is the small amount of sample required for analysis; only nanolitres of sample are introduced into the capillary. Together with high electric fields and short separation lengths, it can produce analysis within seconds. It also allows the metabolic analysis in volume-restricted samples. On the other hand, this leads to low concentration sensitivity requiring enrichment of metabolites within the samples [26]. Another drawback of CE is the poor migration time reproducibility and lack of reference libraries, which may only be partially overcome by the prediction of migration time [27]. Since CE and LC can both separate a large variety of metabolites via fundamentally different mechanisms, they are often used in combination to provide a wider coverage of metabolites [28–30]. That said, the use of CE-MS in plant studies remains relatively rare.

### Nuclear magnetic resonance (NMR) spectroscopy

Nuclear magnetic resonance spectroscopy offers an entirely different analytical technique to that afforded by MS-based techniques being based on atomic interaction. In a strong magnetic field, atoms with non-zero magnetic moment including ^1^H, ^13^C, ^14^N, ^15^N and ^31^P absorb and re-emit electromagnetic radiation. The radiation is characterised by its frequency (chemical shift), intensity, fine structure and magnetic relaxation properties, all of which reflect the precise environment of the detected nucleus. Therefore, atoms in a molecule give a specific spectrum of radiation that can be used for identification and quantification of metabolites within a complex biological sample. The sensitivity of this method is much lower than that of MS-based techniques but the structural information content, reproducibility and quantitative aspects can be superior to them, and some journals require NMR spectra as the final proof of chemical structure [31, 32]. Furthermore the preparation of the sample is simple and even non-destructive measurement is possible. In vivo NMR can further generate kinetic measurements and examine metabolic responses on the same plant rather than on a set of similar plants [33]. The different subcellular pHs of the vacuole from the rest of the cell cause distinctive signals from an identical metabolite and thus allows quantification at the subcellular level [34, 35]. Thus analysing the metabolite composition of a tissue extract, determining the structure of a novel metabolite, demonstrating the existence of a particular metabolic pathway in vivo, isotope labelling experiment and localising the distribution of a metabolite in a tissue are all possible by NMR. For isotope labelling, NMR has the advantage of providing facile access to atomic level labelling, which is highly laborious in the case of MS methods yet can be essential in flux estimation [35]. However, the number of compounds that can be detected in a single analysis is limited to one to several dozen [36, 37]. These properties of NMR make it the ideal tool for broad-range profiling of abundant metabolites whilst studying changes in non-annotated profiles is highly useful for metabolite fingerprinting of extensive sample collections [38, 39].

## Metabolomic studies of plant stress responses

Metabolomics is becoming increasingly common in plant physiology and biochemistry, and to date has been applied to a staggering number of conditions. Here we will attempt a synthesis of the most prominent studies dealing with plant stress; however, the reader is also referred to two previous reviews on this topic [40, 41]. In this section we independently review water stress, temperature stress, light stress, ionic stress, nutrient limitation and oxidative stress before discussing stress combinations. We describe the nature and symptoms of each stress, and then introduce several metabolomic studies with the main metabolic changes observed in each study and the conclusion drawn from the results. Following this survey we discuss commonalities and differences between the various stress responses.

### Water stress

Water limitation is one of the major threats in crop production and this condition is projected to get considerably worse in coming decades [42]. For this reason considerable research effort has been expended to understand the response to this crucial and common stress. These studies have revealed an important role for metabolic regulation including regulation of photosynthesis and accumulation of osmolytes in the drought stress response [43, 44]. Urano et al. [28] reported metabolomic changes in *Arabidopsis* leaves under drought condition. The accumulation of many metabolites was observed, including amino acids such as proline, raffinose family oligosaccharides, γ-amino butyrate (GABA) and tricarboxylic acid (TCA) cycle metabolites, which are known to respond to drought stress in plants. The authors also investigated the *nc3*-*2* mutant, which lacks the *NCED3* gene involved in the dehydration-inducible biosynthesis of abscisic acid (ABA), in order to assess the effect of ABA in the metabolic response to drought stress. By combination with transcriptome analysis they clearly demonstrated that the ABA-dependent transcriptional regulation is responsible to the activation of metabolic pathways including branched chain amino acid, polyamine and proline biosynthesis, GABA shunt and saccharopin metabolism, but is not involved in the regulation of the raffinose biosynthetic pathway during dehydration.

In *Arabidopsis* research, drought tolerance is assessed predominantly under lethal conditions. However, in temperate climates, limited water availability rarely causes plant death but does restrict biomass and seed yield. Results of a recent elegant study experimentally demonstrated that the survival rate under lethal conditions does not predict superior growth performance and biomass yield gain under moderate drought [45], making this mild stress condition more important. Skirycz et al. [46] conducted metabolite profiling of *Arabidopsis* leaves that develop under mild osmotic stress. They revealed that the stress response measured in growing and mature leaves was largely distinct. Typical drought responses, namely accumulation of proline, erythritol and putrecine, were observed only in mature leaves, while many metabolites were decreased in expanding leaves, sharing the same tendency with transcriptional response. When we compared the data from the studies [28, 46], 24 metabolites were detected in both. The decrease of aspartate and increase of proline are the only two responses shared between mildly and severely desiccated leaves. Pronouncedly, amino acid metabolism responds in opposite ways; most amino acids were accumulated in severely desiccated leaves but decreased in mildly desiccated plants. These results highlight the variable response of plant metabolism in different developmental stages and degrees of desiccation. Metabolite profiling has additionally been carried out in crop species exposed to water stress conditions. Intriguingly, common changes in the levels of metabolites including branched chain amino acids were observed in wheat, barley and tomato [47–49].

Too much water, as occurs in situations such as flooding or water-logging of the rhizosphere, also causes problems because of the reduced oxygen availability (hypoxia/anoxia). Under anoxic conditions, ATP has to be produced by fermentation, resulting in cytosolic acidification and the accumulation of toxic products. van Dongen et al. [50] analysed metabolic responses in *Arabidopsis* roots under anoxic conditions. The accumulation of amino acids, alanine, proline and GABA, and the phosphoesters, glucose-6-phosphate and glycerol-3-phosphate, were observed as well as changes in the levels of minor sugars and various organic acids. When oxygen is decreased to 4 %, there is a general tendency for an increase in the levels of the intermediates both of sucrose degradation and the TCA cycle, and in the levels of most amino acids, whereas they are decreased when the oxygen further decreased to 1 %, indicating the inhibition and reactivation of metabolic activities. Together with the transcriptomic data showing a general downregulation of energy-consuming processes, the results demonstrated a large-scale reprogramming of metabolism under oxygen-limited conditions. Rocha et al. [51] examined the accumulation of alanine under anoxic conditions in *Lotus* *japonicus*, which is highly tolerant to water logging. In the roots of *L*. *japonicus*, succinate, alanine and the direct co-substrates for alanine synthesis, glutamate and GABA, were highly accumulated during water logging, whereas the majority of amino acids that are derived from TCA cycle intermediate decreased. The results are in agreement with the metabolic equilibriums that are expected to drive the metabolic flux from glycolysis, via alanine synthesis and oxoglutarate to succinate, which prevents the accumulation of pyruvate activating fermentation and leading to ATP production by succinyl-CoA ligase.

### Temperature stress

Exposure to freezing environments leads to serious damage of the plant cell by ice formation and dysfunction of cellular membranes [52]. Many plant species increase freezing tolerance during exposure to non-freezing low temperature by a process known as “cold acclimation”. The molecular basis of this process has been extensively studied, and the contribution of particular metabolites including compatible solutes [53] and the transcriptional regulatory network has been elucidated [54, 55]. The first metabolomic studies of cold acclimation were performed by two groups in 2004. Cook et al. [56] compared metabolomic changes during cold acclimation in two ecotypes of *Arabidopsis thaliana*, Wassilewskija-2 (Ws-2) and Cape verde islands-1 (Cvi-1), which are relatively freezing tolerant and sensitive, respectively. The metabolome of Ws-2 plants was extensively altered in response to low temperature. Seventy-five percent of metabolites monitored were found to increase in cold-acclimated plants including metabolites known to increase in Arabidopsis plants upon exposure to low temperature, such as the amino acid proline and the sugars glucose, fructose, inositol, galactinol, raffinose and sucrose. They also found novel changes—namely the increase of trehalose, ascorbate, putrescine, citrulline and some TCA cycle intermediates. There was considerable overlap in the metabolite changes that occurred in the two ecotypes in response to low temperature; however, quantitative differences were evident. Kaplan et al. [57] conducted metabolome analysis of *Arabidopsis* over the time course following the shift to cold and heat conditions. Surprisingly the majority of heat shock responses were shared with cold shock including the increase of pool sizes of amino acids derived from pyruvate and oxaloacetate, polyamine precursors and compatible solutes. The results of this study were analysed together with following transcript profiling data by the same group [58], and revealed that the regulation of GABA shunt and proline accumulation under cold conditions are achieved by transcriptional and post-transcriptional manners, respectively. Gray and Heath [59] examined the effects of cold acclimation on the *Arabidopsis* metabolome using a non-targeted metabolic fingerprinting approach. It revealed a global reprogramming of metabolism as well as differential responses between the leaves that shifted to and those that developed in the cold. Hannah et al. [60] took advantage of the natural genetic variation of *Arabidopsis* to elucidate the function of metabolism in cold acclimation. Although there is no clear relationship between global metabolite changes and differences in acclimation capacity or differences between the accessions in acclimated freezing tolerance, the probable importance of central carbohydrate metabolism is indicated by the identification of glucose, fructose and sucrose among metabolites positively correlating to freezing tolerance. Espinoza et al. [61] analysed the effect of diurnal gene/metabolite regulation during cold acclimation by means of metabolomics and transcriptomics. Approximately 30 % of all analysed metabolites showed circadian oscillations in their pool size and low temperature affected the cyclic pattern of metabolite abundance. These results indicated that the interactions observed between circadian and cold regulation are likely highly relevant components of cold acclimation.

Metabolomics was also used to reveal the functions of specific genes in cold acclimation. In the study described above, Cook et al. [56] also investigated plants overexpressing CBF3, which is one of the C-repeat/dehydration responsive element-binding factor (CBF) transcriptional activators induced rapidly under low temperature conditions [62]. The metabolite profiles of non-acclimated CBF3 overexpressing lines were quite similar to those of the cold-acclimated Ws-2 ecotype, suggesting a prominent role for the CBF cold response pathway in configuring the low-temperature metabolome of *Arabidopsis*. Maruyama et al. [63] explored metabolic and transcript changes in *Arabidopsis* plants overexpressing CBF3/dehydration-responsive element binding protein (DREB)1A and another DREB protein DREB2A. They observed similar changes of metabolites in CBF3-overexpressing plants like Cook et al. [56] but DREB2A overexpression showed only a minor effect. The *esk1* mutant is isolated as freezing tolerant without previous acclimation but the function of this gene had been unknown. Lugan et al. [64] tried to elucidate the basis of the freezing tolerance of *esk1* by performing metabolomic analysis under various environmental conditions, namely cold, salinity and dehydration. Then the most specific metabolic responses to cold acclimation were not phenocopied by *esk1* mutation. However, *esk1* accumulated lower amount of Na^+^ in leaves than the wild type and its metabolic profile, and osmotic potential were only slightly impacted under dehydration stress. These results suggested that *ESK1* could rather be involved in water homeostasis and as such highlighted the importance of cellular water status in stress tolerance.

### Light stress

Light is a highly energetic substrate driving photosynthesis that can induce secondary destructive processes at the same time. Therefore, too high light irradiance represents an abiotic stress factor for plants. Wulff-Zottele et al. [65] conducted metabolite profiling of *Arabidopsis* leaves for 6 days after transition to high light. Generally, most of the metabolites of the glycolysis, TCA cycle and oxidative pentose phosphate pathway were altered in their content, indicating that plants exposed to high light undergo a metabolic shift and enhance the Calvin-Benson cycle to fix more carbon. In addition, elevation of glycine indicated the activation of photorespiratory pathways. Caldana et al. [66] investigated the early metabolic response against high light as a part of a more comprehensive study. The accumulation of the photorespiratory intermediates, glycine and glycolate, were observed in the early phase (5–60 min after transition). Interestingly the response during the mid phase (80–360 min) shares similar properties with low temperature treatment, which includes the accumulation of shikimate, phenylalanine and fructose, and the decrease of succinate; however, the physiological meaning of this overlap is currently unknown.

Not only the quantity but also the quality of light affects plant metabolism. In dense plant stands, such as crop fields or forests, individuals shade each other and create competition for light absorption [67]. Selective light absorption by the upper leaf layers leads to an enrichment of far-red wavelength [68], which induces excitation imbalances between photosystem II and I disturbing both the redox chemistry in the transport chain and its coordination with the Calvin-Benson cycle [69, 70]. For this reason, Bräutigam et al. [71] grew *Arabidopsis* plants under light, which preferentially excited either photosystem I (PSI light) or II (PSII light) and then transferred it to the other light condition to analyse how plants acclimate to the light quality shift. After long-term acclimation of 48 h, plants exhibited two distinct metabolic states. A PSI–II shift resulted in a decrease in primary products of photosynthesis, such as sugars, but an increase in important intermediates of subsequent metabolic pathways. By contrast, a PSII-I shift has no effect on the sugar pools but leads to general downregulation of many subsequent metabolites, including amino acids and organic acids. Each of the metabolites exhibited a different accumulation profile for establishing the final pool size, indicating high complexity by which the two metabolic states were achieved. Comprehensive analyses of these data alongside transcript profiles and other physiological data suggested that photosynthesis and metabolism were under the control of a binary combination of inputs from the thioredoxin and plastoquinone systems.

The dependency of plants upon sunlight also inevitably leads them into exposure to ultraviolet (UV) light, including in the wavelength range of 280–320 nm (UV-B). This wavelength potentially damages DNA, RNA and proteins, and additionally increases the production of free radicals [72, 73]. Kusano et al. [74] treated *Arabidopsis* plants with UV light and analysed the metabolic effect of UV light stress. Arabidopsis exhibits an apparent biphasic response to UV-B stress, characterised by major changes in the levels of primary metabolites, including ascorbate derivatives. By contrast, mid- to late-term responses were observed in the classically defined UV-B protectants, such as flavonoids and phenolics. The results suggested that in early stages of exposure to UV-B, the plant cell is ‘primed’ at the level of primary metabolism by a mechanism that involves reprogramming of the metabolism to efficiently divert carbon towards the aromatic amino acid precursors of the phenylpropanoid pathway. It also suggested the importance of ascorbate in the short-term response to UV-B. Further studies are, however, required to determine which of these metabolic changes are end responses to adapt to the enhanced exposure to UV-B and which are part of the perception-signalling relay, which alerts the plant cell that it needs to respond to the stress [75].

### Ion stress

High levels of salinity in the soil hinder the growth and development of crops and cause serious problems for world food production [76]. High concentrations of NaCl may cause both hyperionic and hyperosmotic stress effects, which lead to a decline of turgor, disordered metabolism and the inhibition of uptake of essential ions, as well as other problems in plant cells [77, 78]. Gong et al. [79] conducted metabolite profiling of salt-treated *Arabidopsis thaliana* and its relative *Thellungiella* *halophila* (salt cress), which shows ‘extremophile’ characteristics manifested by extreme tolerance to a variety of abiotic stresses, among them low humidity, freezing and high salinity. Proline increased dramatically in both species as did inositols, hexoses and complex sugars. The concentrations of metabolites were often several-fold higher in *Thellungiella* and stress exacerbated the differences in some metabolites. Transcript analyses supported the metabolic results by suggesting that a *Thellungiella* is primed to anticipate such stresses. The difference in metabolites between *Arabidopsis* and *Thellungiella* under salt and osmotic stresses was more recently assessed for a broader range of metabolites [80]. Analysis of global physicochemical properties of metabolites revealed a shift from nonpolar to polar metabolites in both species but that this was much more pronounced in *Thellungiella*. Such a shift may contribute to keep the water potential during dehydration. Kim et al. [77] investigated the cellular level metabolic response using *Arabidopsis* T87 cultured cells. The results suggested that the methylation cycle for the supply of methyl groups, the phenylpropanoid pathway for lignin production and glycine betaine biosynthesis are synergetically induced as a short-term response against salt-stress treatment. The results also suggest the co-induction of glycolysis and sucrose metabolism as well as co-reduction of the methylation cycle as long-term responses to salt stress.

Due to the importance of salinity stress in agriculture, there are many metabolomic studies to assess the metabolic effect of salinity in a variety of crop and related plant species including tomato [40, 81], grapevine [82], poplar [83], sea lavender (*Limonium latifolium*, [84]) and rice [85]. Since these studies have been extensively reviewed in [40, 86], we focus here on three recent studies on legume species [87–89]. These recent studies took a functional genomic approach that integrated ionomic, transcriptomic and metabolomic analyses of the glycopyte model legume *Lotus japonicus* and other *Lotus* species subjected to long-term regimes of non-lethal levels of salinity. In *Lotus japonicus* the metabolic changes were characterised by a general increase in the steady-state levels of many amino acids, sugars and polyols, with a concurrent decrease in most organic acids [87]. The responses to salinity stress were compared between extremophile (*L*. *creticus*) and glycophytic (*L*. *corniculatus* and *L*. *tenuis*), but the metabolic responses were globally similar to each other [88]. These results suggest that, in contrast to *Thellungiella*, the metabolic pre-adaptation to salinity is not the major trait of *L. creticus* contributing to the extramophile phenotype. However, by comparing six species displaying different salt tolerances, they observed several genotype-specific features. One of them is the increase of asparagine levels in the more tolerant genotypes, suggesting that the roles of asparagine metabolism in supporting core nitrogen metabolism may play a role in tolerance [89].

Heavy metals such as cadmium (Cd), cesium (Cs), lead (Pb), zinc (Zn), nickel (Ni) and chromium (Cr) are major pollutants of the soil causing stress on plants. Even the essential nutrients including copper (Cu), iron (Fe) and manganese (Mn) can cause heavy metal stresses with inappropriate concentration. Generally heavy metals induce enzyme inhibition, cellular oxidation and metabolic perturbation, resulting in growth retardation and in extreme instances in plant death [90]. Jahangir et al. [91] analysed the effects of Cu, Fe and Mn on the metabolite levels of *Brassica* *rapa*, which is a known metal accumulator. Glucosinolates and hydroxycinnamic acids conjugated with malates as well as primary metabolites such as carbohydrates and amino acids were found to be the discriminating metabolites. *Arabidopsis* plants treated with Cd displayed increased levels of alanine, β-alanine, proline, serine, putrescine, sucrose and other metabolites with compatible solute-like properties, notably GABA, raffinose and trehalose [92]. This study also indicated that concentrations of antioxidants (α-tocopherol, campesterol, ß-sitosterol and isoflavone) also increased significantly. When taken together these data indicate an important role of antioxidant defences in the mechanisms of resistance to cadmium stress. Dubey et al. [93] conducted transcriptomic and metabolomic analysis of rice roots treated with Cr. Under these conditions proline accumulated to levels three-fold those of the control as did ornithine, which can be used in its synthesis. The content of several other metabolites including lactate, fructose, uracil and alanine increased following exposure to Cr stress; these were taken to suggest the modulation of the sucrose degradation pathway involving the three main fermentation pathways operating as a rescue mechanism when respiration is arrested. Further studies are however most likely warranted to gain a better understanding of the mechanisms underlying these changes.

### Nutrient limitation

Nutrient starvation also dramatically affects plant growth and metabolism. Especially limitation of macronutrients, namely carbon (C), nitrogen (N), phosphorus (P) and sulphur (S), has direct effects on metabolism since most organic molecules comprise a combination of these elements. Changing environmental conditions continually alter the balance between C assimilation and utilisation. Even short periods of C starvation lead to an inhibition of growth, which is not immediately reversed when C becomes available again [94, 95]. Osuna et al. [96] investigated the metabolite profile of *Arabidopsis* seedlings in liquid culture under C starvation. In C-starved seedlings, as could be anticipated, carbohydrates, organic acids and other C-containing metabolites, including *myo*-inositol, raffinose, glycerate and fatty acids, decreased. Central amino acids (glutamine, glutamate, aspartate and alanine) and methionine, an S-containing amino acid, also decreased, indicating the inhibition of N and S assimilation, respectively. The increase of most other amino acids indicates that proteolysis has commenced. Most of these changes reverted rapidly after re-addition of sucrose into the media. Usadel et al. [97] took advantage of extended dark treatment to induce C starvation under more natural conditions in the *Arabidopsis* rosettes. Intriguingly, however, the changes in metabolite levels were mostly comparable to those observed in liquid culture seedlings [96]. The marked decrease of carbohydrates within the first 4 h of extended night indicates that the treatment induced C starvation very efficiently and that carbohydrates are starting to acutely limit metabolism. On the other hand, organic acids and other C-containing metabolites displayed a rather gradual decrease. The prolonged dark treatment induced severe C starvation and leaf senescence by the end of the experiment. The metabolite profile of *Arabidopsis* leaves subjected to prolonged darkness has been analysed in a series of studies to elucidate the metabolic bases of dark-induced senescence and the function of the mitochondrial alternative electron transport pathway during dark treatment [98–100]. Although a similar metabolic phenotype as the two studies described above [96, 97] was observed during the first few days of dark treatment, a subset of metabolites exhibits biphasic behaviour during prolonged exposure to darkness. This was particularly notable for some TCA cycle intermediates including fumarate, isocitrate, malate and succinate, which accumulated after 7 days of dark treatment despite decreasing during the first 3 days of treatment. Additionally accumulation of most amino acids including GABA became much more prominent. Metabolite profiles were also analysed in a range of mutants deficient in the genes involved in mitochondrial alternative electron transport mediated by the electron-transfer flavoprotein/electron-transfer flavoprotein:ubiquinone oxidoreductase (ETF/ETFQO) complex, namely *ETFQO* [98] and *ETFβ* [99] as well as enzymes involved in the provision of its substrates, namely *IVDH*, *D2HGDH* [101] and *PSHX* [100]. Although individual genotypes showed similar responses during the first 3 days of dark treatment, there are subtle differences in their metabolic complements at the end of the experiment, indicating an essential role of this alternative electron transport machinery during dark-induced starvation [99]. Further detailed analysis revealed that the ETF/ETFQO complex is involved in both the branched chain amino acids and the lysine catabolism pathways, and acts as an electron donor to the mitochondrial ubiquinol pool [100, 101]. These studies suggest that more integrative analysis of the role of all aspects of protein degradation and consequent remobilisation should be performed within the context of understanding metabolic responses to stress.

Nitrogen is required for the synthesis of nucleotides and amino acids, which are the building blocks of nucleic acids and proteins, and for the synthesis of phospholipids and many secondary metabolites that have diverse roles in signalling, structure and adaptation. The effect of N deficiency on the metabolite levels in tomato leaves were investigated by Urbanczyk-Wochniak and Fernie [102]. As would perhaps be expected, amino acid levels generally decreased under nitrogen deficiency. The level of 2-oxoglutarate, a key regulator of carbon and nitrogen interactions [103], decreased under N starvation as well as other TCA cycle intermediates including citrate, isocitrate, succinate, fumarate and malate. Tschoep et al. [104] analysed the effect of mild but sustained N limitation in *Arabidopsis*. Malate and fumarate levels were strongly decreased in low N conditions like in tomato leaves [102]. However, their rosette protein content was unaltered and total, and many individual amino acid levels increased compared with N-replete plants. The results revealed that *Arabidopsis* responds adaptively to low N condition. P is an essential component of intermediates in central and energy metabolism, signalling molecules and structural macromolecules like nucleic acids and phospholipids. Morcuende et al. [105] analysed the metabolite profile of *Arabidopsis* seedlings grown in liquid culture under P starvation. The levels of sugar phosphates were very low but metabolites further down in glycolysis, glycerate-3-phosphate, glycerate-2-phosphate and phosphoenolpyruvate, increased in P-deficient seedlings. Pi-deficient seedlings showed a marked accumulation of starch, sucrose and reducing sugars as well as a general increase of organic acids including citrate, fumarate, malate and oxoglutarate. The levels of most major amino acids did not alter or increased slightly, whereas those of several minor amino acids including the aromatic amino acids and histidine, arginine and threonine. Together with transcriptomic data, analysis of metabolites revealed that P deprivation leads to a shift towards the accumulation of carbohydrates, organic acids and amino acids. The effect of P starvation has also been studied on crop plants such as common bean and barley. Hernández et al. used metabolite profiling to assess the effect of P deficiency in the roots [106] and nodules [107] of the common bean. Most of the amino acids were increased in P-stressed roots. The accumulation of several sugars suggests that sugars may be partitioned preferentially to P-stressed roots to support the expression of P stress-induced genes. The reduced amounts of organic acids likely reflect exudation of these metabolites from the roots into the rhizosphere [106]. The metabolic response of P-starved nodules is in contrast to that of roots. Amino acids and other N-containing metabolites were decreased as well as sugars, while organic acids were accumulated in P-deficient nodules. Such a contrasting response may be due to the N deficiency in P-starved nodules in which the sole N supply from fixed N_2_ could be suppressed under environmental limitations such as P starvation [107]. Huang et al. [108] profiled metabolites from both shoots and roots of P-deficient barley. Severe P deficiency increased the levels of phosphorylated intermediates (glucose-6-P, fructose-6-P, inositol-1-P and glycerol-3-P) and organic acids (2-oxoglutarate, succinate, fumarate and malate). The results revealed that P-deficient plants modify carbohydrate metabolism initially to reduce P consumption and salvage P from small P-containing metabolites, which consequently reduce the levels of organic acid in the TCA cycle [108].

Sulphur is another macronutrient essential for the synthesis of the S-containing amino acids cysteine and methionine as well as a wide range of S-containing metabolites including glutathione. There are some metabolomic studies on the response to S starvation in *Arabidopsis* [109–112], and they are nicely summarised in Hoefgen et al. [113]. At the time course of S-stress response, two metabolic states can be distinguished. The short-term metabolic responses include the decrease of organic S-containing compounds on the S assimilation such as cysteine and glutathione, which leads to the accumulation of their precursor *O*-acetyl-serine (OAS) as well as serine, and to the subsequent re-channeling of the metabolic flow to glycine and tryptophan. Glucosinolate catabolism is activated to salvage S from it. As a long-term response the lipid contents and a S-containing molecule, *S*-adenosyl-methionine, decreased. Insufficient S supply leads to its disbalance with N and further to the alterations in C1 metabolism that link photorespiration, S assimilation and dumping of N [113]. Results of a very recent study on the *Arabidopsis* plants with modified OAS levels suggest the importance of this metabolite since OAS plays a signalling role for a specific part of the sulphate response as well as for the regulation of the transcript levels of a specific gene set irrespective of the sulphur status of the plants [114].

Potassium (K) is not a component of organic molecules but plays essential roles as a major cation in plants and as a cofactor of enzymes [115]. Armengaud et al. [116] used metabolite profiling to identify metabolic targets of K stress. Metabolite profiles of low-K *Arabidopsis* plants were characterised by a strong increase in the concentrations of soluble sugars (sucrose, fructose and glucose) and a slight net increase of total protein content and the overall amino acid level. Several basic or neutral amino acids accumulated during K deficiency, while acidic amino acids decreased. In addition a strong decrease of pyruvate and organic acids was recorded only in the roots but not in the shoots. They also measured enzyme activities and concluded that the primary effect of K deficiency induces an inhibition of glycolysis by the direct inhibition of enzymes [116].

### Oxidative stress

Oxidative stress is a key underlying component of most abiotic stresses and a major limiting factor of plant growth in the field [117]. It occurs on the overproduction of reactive oxygen species (ROS) in plant cells when plant metabolism is perturbed by various stresses. This consequently leads to oxidative damages of cellular components such as DNA, proteins and lipids [118]. To cope with oxidative stress, the metabolic network of plant cells must be reconfigured either to bypass damaged enzymes or to support adaptive responses. In the study by Baxter et al. [119], heterotrophic *Arabidopsis* cells were treated with menadione, which enhances the ROS production via electron transport chains and changes in metabolite abundance, and ^13^C-labelling kinetics were quantified. The accumulation of sugar phosphates related to glycolysis and oxidative pentose phosphate pathways (OPPP) suggested the rerouting of glycolytic carbon flow into the OPPP possibly to provide NADPH for antioxidative effort. In addition the decrease of ascorbate and accumulation of its degradation product, threonate, indicated the activation of antioxidative pathways in menadione-treated cells. The reduced glycolytic activity probably leads to the decrease of levels of amino acids derived from glycolytic intermediates. The decrease of amino acids linked to TCA cycle intermediates and decrease of malate indicated a perturbation of TCA cycle. These observations in metabolite levels were emphasised by ^13^C-redistribution analysis, which indicated increased carbon flux into OPPP intermediates and inhibition of metabolic flux into all TCA cycle intermediates detected [119]. Lehmann et al. [120, 121] also conducted both metabolite profiling and ^13^C-redistribution analysis of menadione-treated *Arabidopsis* roots and found that the metabolic response of roots is distinct from that of heterotrophic cells in culture [120]. The redirection of glycolytic carbon flow and inhibition of the TCA cycle were suggested also in the roots. Especially the inhibition of the TCA cycle is more evident in roots as a perturbation of metabolite levels. In addition, roots showed pronounced accumulation of some metabolites including GABA, OAS, pyruvate, many amino acids and glucosinolates. It seems likely that cellular oxidation inhibited S assimilation and caused OAS accumulation. A general increase of amino acid levels is thought to be the result of enhanced protein degradation. This is supported by ^13^C-labelling analysis in which the ^13^C-redistribution was not affected in most amino acids, indicating that the carbon in the increased amino acids was not from synthetic pathways [121]. They also followed the metabolic recovery process after the removal of menadione from the culture media [121]. After menadione removal many of the stress-related changes reverted back to basal levels. However, each metabolic pathway recovered in a differential time period, for instance, glycolytic carbon flow reverted to control level 18 h after menadione removal, although the TCA cycle and some amino acids such as aspartate and glutamate took longer to recover. It suggests the involvement of pathway-specific regulatory processes for the oxidative stress response. These metabolic responses to menadione-induced oxidative stress mentioned above seem to be conserved among plant species and organs because quite similar responses were observed both in *Arabidopsis* seedlings in liquid culture [122] and rice suspension cells [123]. They are additionally at least partially similar to those observed when oxidative stress is mimicked by the removal of enzymes involved in ameliorating against it, such as manganese superoxide dismutase [124]. In the *Arabidopsis* plants with suppressed expression of mitochondrial manganese superoxide dismutase revealed a decrease of TCA cycle intermediates, probably because of the inhibition of aconitase and isocitrate dehydrogenase [124].

### Stress combination

Whilst convenient both for experiments and discussion at the single stress level, plants are actually subjected to a combination of abiotic stress conditions in their natural habitat. Even some abiotic stresses are already combinations of stresses. For example high salt concentration causes osmotic and ion stresses, and flooding results in hypoxic and shading stresses. Although the metabolic responses of plants under a single abiotic stress have been analysed extensively as shown above, there are only few studies regarding to the effect of stress combinations on plant metabolism. Rizhsky et al. [125] applied a combination of drought and heat stress to *Arabidopsis* plants and analysed the metabolic profile. The metabolite profile of plants subjected to a combination of drought and heat stress was more similar to that of plants subjected to drought than to that of control plants or plants subjected to heat stress. However, the plants subjected to combined stresses accumulated high levels of sucrose and other sugars instead of proline, which is highly accumulated to a very high level in plants subjected to drought but not under stress combination. They concluded that sucrose replaces proline as the major osmoprotectant in plants subjected to combined stress because the toxic effect of high level of proline is enhanced under heat stress, as they showed experimentally [125]. Wulff-Zottele et al. [65] analysed the effect of the combination of high light irradiance and S depletion, which can occur in the field simultaneously [126]. The combination of high light and S depletion gives rise to similar metabolic pool modifications such as in high light. Proline accumulated in a differential time course under high light and stress combination. Other metabolites such as raffinose and putrescine seem to replace proline during the delay of proline accumulation in the plants subjected to high light and S depletion. This replacement of proline with sugars is similar to that observed under the combination of drought and heat stress [125]. Recently Caldana et al. [66] reported a systematic study on the metabolomic and transcriptomic response of *Arabidopsis* to eight environmental conditions including the combinations of changing light (darkness, high light) and/or temperature (cold and heat). The analysis has demonstrated that darkness and high temperature have a synergistic effect, thus presenting a more extreme condition. The reconstructed metabolic networks for this condition also revealed an exclusive correlation between several amino acids, including GABA with intermediates of the TCA cycle, notably succinate. These results suggested that in the absence of photosynthesis protein degradation occurs rapidly and subsequent amino acid catabolism serves as the main cellular energy supply [66].

## Common and stress-specific metabolic responses against diverse abiotic stress

**Fig. 1:**
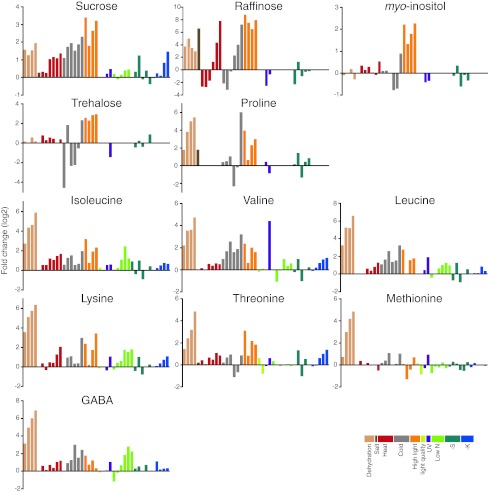
Changes of the levels of metabolites in *Arabidopsis* leaves under various abiotic stress conditions. Each datum represents the relative metabolite level in the fold change value against control growth conditions at one time point. The values are taken from studies on dehydration [28], salt [79], heat and cold [57], high light [65], light quality change [71], UV-B light (UV) [74], low nitrogen (Low N) [104], sulphur limitation (-S) [65] and potassium limitation (-K) [116] stresses. The data set used for the analysis is found in Supplementary data, Table S1. The *bars* with different colours represent the values from different studies as shown in the figure. Only the metabolites of interest are shown. The *charts* for all metabolites are presented as Supplementary data, Fig. S1

**Fig. 2:**
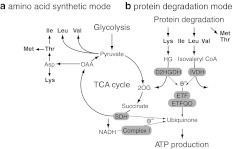
Metabolic modes leading to the accumulation of branched chain amino acids (BCAA) and related amino acids suggested under abiotic stress conditions. **a** Amino acid synthetic mode. BCAAs are synthesised using pyruvate or oxaloacetate (OAA) as carbon skeletons. **b** Protein degradation mode. Amino acids resulting from degraded proteins would be direct and indirect electron donors to produce ATP. *2OG* 2-oxoglutarate, *SDH* succinate dehydrogenase, *HG* hydroxyglutarate, *D2HGDH* d-2-hydroxyglutarate dehydrogenase, *IVDH* isovaleryl-CoA dehydrogenase, *ETF* electron transfer flavoprotein, *ETFQO* ETF-ubiquinone oxidoreductase, *e* ^−^ electron

As described above, plants show a variety of metabolic responses against diverse abiotic stresses. The question is whether there are any common metabolic responses to all abiotic stresses or the responses are always specific to the stress factors. To evaluate the accumulation of these compounds under stress conditions and to search for the novel metabolic fingerprints related to the stress responses, we analysed the published metabolite profiling data available in the above-mentioned literature. Studies dealing with *Arabidopsis* leaves were chosen (dehydration [28], salt [79], heat and cold [57], high light and sulphur limitation [65], UV [74], light quality change [71], low nitrogen [104] and potassium limitation [104]) to afford greater comparability. Forty-five metabolites detected in more than half of the studies were analysed and each datum was converted into the fold change values against control growth conditions and presented using the log_2_ scale (Supplementary data, Table S1). Table 1 shows the number of metabolites accumulated or decreased under each stress condition. This reveals that abiotic stresses generally induce accumulation of metabolites with only the light quality change as an exception. The tendency of accumulation is probably related to a cessation of the growth-reducing consumption of metabolites. When subjected to abiotic stresses, plants actively re-program their growth by modulating both cell division and cell expansion. Growth decreases rapidly upon stress onset, but it recovers and adapts once stress conditions become stable [127]. Accumulated metabolites might be used as building blocks to support a recovery of growth. Figure 1 provides an overview of the changes in the amount of selected metabolites. Charts for all metabolites are found in the Supplementary data, Fig. S1. In general, changes in the amounts of metabolites were stress-specific in contrast to the general responses observed in bacteria [128]. A stress-specific change in the metabolite level would be a result of an inhibition/activation of a specific metabolic pathway especially in the short term. It should mainly be related to the properties of enzymes such as sensitivity to temperature, oxidation and ion concentration. In addition, rearrangement of the metabolic network should also result in changes of metabolites, which are related to the regulated pathways. Therefore, such a metabolite must be a good candidate for an analysis to elucidate the specific effects of an abiotic stress and the adaptive processes against it. On the other hand, metabolites responding to various stresses can be related to fundamental stress responses. In the present analysis, some metabolites can be seen to accumulate in most abiotic stress conditions although the time and extent of accumulation varied among conditions. Levels of sucrose were increased in most stress conditions in at least one time point (Fig. 1). Sucrose is a major transport sugar in most plant species and is known to accumulate under stress conditions [129]. Compounds defined as “compatible solutes” also accumulate under various abiotic stress conditions. They are very soluble in water and are non-toxic at high concentrations and function to sustain the ordered vicinal water around proteins by decreasing protein-solvent interactions at low water activities [52, 130]. This group of compounds includes betaines and related compounds; polyols and sugars, such as mannitol, sorbitol and trehalose; and amino acids, such as proline [131, 132]. Recent studies have revealed that they function to protect plants not only from osmotic stress but also from various stress factors [130, 131, 133, 134]. Therefore, the synthetic pathways of those metabolites have been of interest for metabolic engineering and some interventions have indeed increased the tolerance of some crop plants to abiotic stress [130, 131]. Raffinose is a sugar synthesised from sucrose and known to protect plant cells as an osmoprotectant; it also accumulates under most stress conditions especially at the later stages of stress treatment (Fig. 1). Raffinose is also shown to function to protect plants from oxidative damage [135], making the observation reasonable since oxidative damage likely underlies most stress conditions. Interestingly, *myo*-inositol, which is closely related to raffinose biosynthesis, did not show prominent changes other than under high light conditions (Fig. 1). The amino acid, proline, is known as a major compatible solute in *Arabidopsis* [133] and also accumulated under stress conditions despite being detected only in a limited number of studies (Fig. 1). On the other hand, trehalose accumulated only under specific conditions, suggesting that it displays functions other than being a compatible solute (Fig. 1). Indeed it is unlikely that trehalose contents in plants—other than resurrection plants—are high enough to be directly involved in stress protection [136] and some trehalose metabolism mutants exhibit potential negative effects on plant physiology [134]. The amount of trehalose may reflect that of its precursor, trehalose-6-phosphate, which has been documented to act as a signal molecule in plants [137]. GABA is another metabolite discussed in a context of stress response since it is largely and rapidly produced in response to biotic and abiotic stresses [138–140]. Our analysis supported this observation (Fig. 1). There are many suggested functions of GABA and the GABA shunt, which protect plants to survive various stress conditions including regulation of cytosolic pH, protection against oxidative stress and functions of GABA as an osmoregulator and as a signalling molecule [138]. However, whilst evidence for an important metabolic role has been documented, that for a signalling role in plants is still lacking. In the presented data set, branched chain amino acids (BCAAs), namely valine, leucine and isoleucine, and other amino acids sharing synthetic pathways with BCAA, including lysine, threonine and methionine (Fig. 2a), were generally accumulated under abiotic stress conditions. These amino acids are a novel group of metabolites that accumulated generally in response to stress conditions, although they have been shown to accumulate under drought stress conditions [141]. Joshi et al. [141] suggested that they function as compatible osmolytes since BCAA showed a high fold increase under drought stress in various plant tissues. Another possible role of BCAAs under stress conditions would be that of an alternative electron donor for the mitochondrial electron transport chain. The mitochondrial electron transport chain is primary supplied by electrons from NADH and succinate to produce ATP. Additionally there is an alternative way to feed electrons from other substrates via electron transfer flavoprotein (ETF) complex. Recent studies highlighted the importance of the alternative pathway under dark and stress conditions especially under carbon starvation (see also the "Nutrient limitation" section) [98–101]. A ^13^C-feeding experiment has proven that lysine and BCAA are converted into d-2-hydroxyglutarate and isovaleryl-CoA in vivo to be a direct electron donor for the ETF complex via the action of d-2-hydroxyglutarate dehydrogenase and isovaleryl-CoA dehydrogenase [101]. As such, BCAAs can provide electrons both directly to the electron transport chain via the ETF complex as well as indirectly because their catabolic products feed directly into the tricarboxylic acid (TCA) cycle [142] (Fig. 2b). The source of accumulated BCAAs would be the protein degradation product, which has recently been proposed to be an important alternative respiratory substrate especially under certain stress conditions [142], as well as the activated synthetic pathway, which is observed under drought stress conditions [28, 141]. Thus, our analysis emphasised the importance of BCAA metabolism generally under abiotic stress conditions. Interestingly, the pattern of accumulation of GABA is similar to those of BCAAs (Fig. 1), although the reason why they are related under stress conditions remains unclear. Thus, clarifying the exact mechanistic role of BCAAs under various conditions will be an important priority for the future.

**Table 1:** Number of metabolites that changed their abundance under each stress condition

Condition	Increased	Decreased
Dehydration	26	2
Salt	3	0
Heat	10	4
Cold	27	9
High light	39	4
Light quality	1	7
UV	14	5
Low N	10	3
-S	11	2
-K	13	0

The changes greater than two fold were counted

## Prospective: toward the elucidation of molecular mechanisms underlying abiotic stress tolerance

A wealth of metabolomics data concerning the plant stress response has been accumulated and a large number of metabolic pathways are suggested to be regulated under stress. However, there are relatively few pathways and metabolites that have been experimentally proven to function in abiotic stress tolerance. One problem is that a metabolite profile does not tell exactly whether the related metabolic pathway is up- or downregulated since both upregulation of upstream reaction and down-regulation of downstream reactions can lead to the accumulation of a metabolite. This can be solved by comparing the metabolomic data with those from transcriptomic or proteomic analysis or activities of specific enzymes [143]. Hirai et al. [111] revealed gene to metabolite regulatory networks of glucosinolate synthesis and primary metabolism under sulphur- and nitrogen-limited conditions by applying integrated analysis of transcriptome and metabolome data. Integrated analyses of the transcriptome and the metabolome successfully demonstrated connections between genes and metabolites, elucidating a wide range of signal output from ABA under dehydration [28] and the DREB1/CBF transcription factors in response to low temperature [63] as described above. This approach is proven to be useful to elucidate the regulation of the pathway and also the involvement of transcriptional regulation of the pathway. The studies using proteomics together with metabolomics are relatively rare in the plant stress response field. One example is the study by Wienkoop et al. [144], which showed the importance of starch and raffinose family oligosaccharide metabolism during temperature stress by the metabolomic and proteomic analysis of the starch-deficient *Arabidopsis* mutant lacking phosphoglucomutase (*pgm* mutant). The number of such studies should increase in the near future because of the improvement of analytical methods for proteomics. The activities of enzymes involved in a pathway should have a direct relationship with the amount of a metabolite and could be a useful tool to assess the metabolic regulation. Changes in maximal enzyme activities were analysed together with transcriptomic and metabolomic data in the study by Armengaud et al. [116], which pinpointed that pyruvate kinase activity was inhibited directly by K deficiency and was primarily responsible for the metabolic disorders observed. Metabolic flux analysis is another powerful approach to study the regulation of metabolic pathways. Lehmann et al. [121] conducted ^13^C redistribution analysis to prove the downregulation of glycolysis under oxidative stress treatment, which is suggested by metabolic profiling [120]. Metabolic flux analysis is also useful to elucidate a metabolic regulation that cannot be detected by metabolic profiling since the carbon flow can be affected without any apparent changes in the metabolite pool sizes [119, 145, 146]. Improvement of both experimental and theoretical mathematical approaches to flux estimation and parameterisation will greatly aid our understanding here [147]. The analysis of cell-type-specific responses would reveal more detailed mechanisms of the stress response that are hidden in a mixture of the cells in a tissue. Although the fluorescence-activated cell sorting (FACS)-based transcriptome and immunoprecipitation-based translatome data sets have provided an important foundation for the analysis of the transcriptional and translational control of environmental responses in each tissue layer of the plant [148, 149], the metabolomic studies are still rare because of the technical difficulties. Ebert et al. [150] applied single cell sampling using microcapillaries to enable the cell-type-specific metabolic analysis of epidermal cell types in *Arabidopsis thaliana* pavement, basal and trichome cells. Recently, Rogers et al. [151] demonstrated the feasibility of FACS-based metabolic profiling using high-resolution mass spectrometry at cell type resolution in roots.

The integration of the “omics” data revealed many molecular mechanisms for metabolic regulation but also highlighted a complex relationship among the levels of transcripts, metabolites and metabolic flux. It suggests the participation of post-transcriptional especially post-translational regulation of enzyme activity in the regulation of primary metabolism. An important role of post-transcriptional regulation in the stress response is also suggested by the poor statistical correlation of protein expression data with microarray results especially in the short-term response [152–156]. And there is an explicit indication that considerable metabolic control is executed on the metabolite and on the protein level including protein modifications [157]. Metabolic enzymes are well known to be regulated allosterically by the substrates and/or the products of the pathway [158, 159]. Many other post-translational modifications of the enzyme proteins such as phosphorylation, glutathionylation and nitrosylation could be involved in metabolic regulation [160–162]. Among them, we discuss here the reconfiguration of enzyme protein complexes in this study. In the protein complex “substrate channeling” can happen by which the intermediate produced by one enzyme is transferred to the next enzyme without complete mixing with the bulk phase [163]. Especially the association of several sequential metabolic enzymes involved in one pathway is called a metabolon [164]. Metabolite channeling can be envisioned as a means to improve catalytic efficiency by increasing local substrate concentrations, regulating competition between branch pathways for common metabolites, coordinating the activities of pathways with shared enzymes or intermediates, and sequestering reactive or toxic intermediates [165, 166]. The organisation of metabolic pathways by metabolic channeling has been discussed as the main molecular-scale organisation units to orchestrate the multiple metabolic processes and it is now supported by modelling as well as experimental evidence [167–170]. Recent bioinformatic study suggested that evolved protein interactions may contribute significantly towards increasing the efficiency of metabolic processes by permitting higher metabolic fluxes [171]. Metabolite channeling is considered to be achieved not only when the enzyme association is stable but also when the association is dynamic. Transient complexes offer the possibility of fast exchange of some of the polypeptide components upon reassembly and thus can be a molecular basis for rapid fine tuning or redirection of metabolism. The reassembly of the metabolic enzyme complex therefore should have a molecular basis underlying the metabolic regulation in mitochondria under short-term oxidative stress. Metabolic channeling including metabolon formation is reported in many processes in plants such as glycolysis, cysteine synthesis, the Calvin-Benson cycle, cyanogenic glucoside biosynthesis, the phenylpropanoid pathway, the glycine decarboxylase system and polyamine biosynthesis [165, 166]. There is however little evidence showing metabolic channeling in plant mitochondrial metabolism although the metabolon of TCA cycle enzymes and enzymes involved in amino acid metabolism are well documented in bacteria, yeast and mammals [164, 168]. More plant proteins forming enzyme super complexes should exist and contribute to metabolic regulation. Our recent study suggested that some metabolic enzymes, including malic enzyme and alanine amino transferase, altered their status in a protein complex in relation to metabolic regulation under an oxidative stress condition [122]. Further analysis is necessary to prove the involvement of enzyme protein interactions in metabolic regulation under abiotic stress conditions. Nevertheless, the expected results would lead to the new insight into plant metabolic regulation and to the full understanding of the metabolic events under abiotic stress conditions and further for the breeding of stress-tolerant crops by elucidating the target metabolic pathways to be modified.

To summarise, experiments to date have allowed us to catalogue a vast array of metabolic changes in response to stress. Without overgeneralising, since some of these are very well understood at a mechanistic level, our understanding of the causes and effects of these changes remains in some cases rather fragmentary. The metabolic changes in stress responses are considered to be divided into three phases, including a direct effect of environmental changes, transient adaptation to stress conditions and the new steady state established under prolonged stress conditions. It should be noted that each phase adopts a different duration depending on the type and the severity of the stress. A detailed time course experiment, such as those conducted in [66] or [128], is therefore necessary to distinguish to which phase the metabolic changes are related. It is also highly likely that integrating the results already obtained with those from isotope feeding experiments, comprehensive phytohormone measurements and further transcriptomic and proteomic studies will further deepen our understanding of these crucial survival processes. Once obtained such information will provide an immense foundation for metabolic engineering and synthetic biology approaches to ensuring food security.

## Electronic supplementary material

Below is the link to the electronic supplementary material.

Supplementary material 1 (PPT 302 kb)
