# Supplementary material for: The use of metabolomics to dissect plant responses to abiotic stresses
Source: Cell Mol Life Sci. 2012 Aug 12;69(19):3225–43. doi: 10.1007/s00018-012-1091-5 (PMC3437017; doi:10.1007/s00018-012-1091-5)
Supplement: Supplementary file 1 — Supplementary material 1 (PPT 302 kb) [file 18_2012_1091_MOESM1_ESM.ppt]

## Slide 1
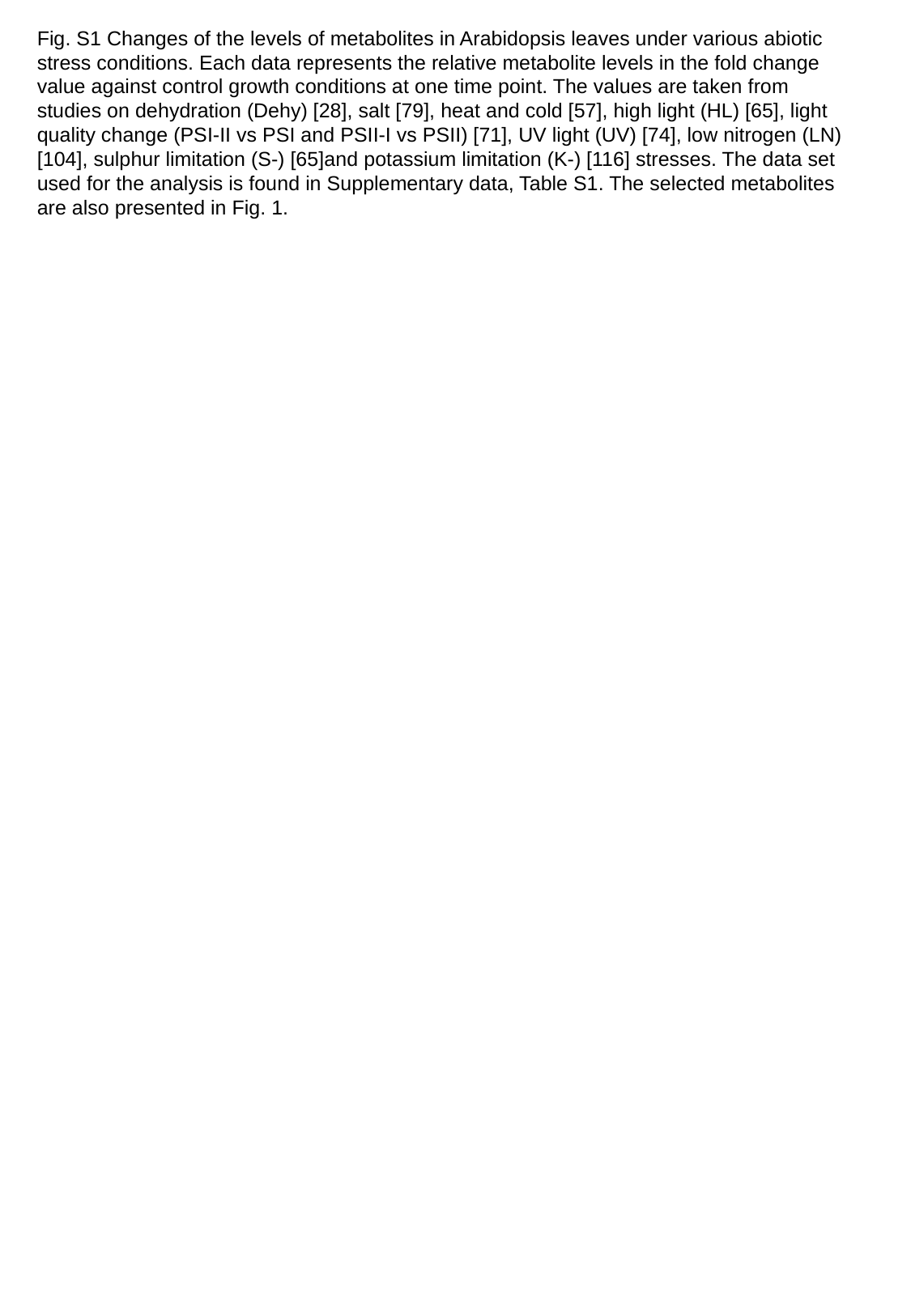

Fig. S1 Changes of the levels of metabolites in Arabidopsis leaves under various abiotic stress conditions. Each data represents the relative metabolite levels in the fold change value against control growth conditions at one time point. The values are taken from studies on dehydration (Dehy) [28], salt [79], heat and cold [57], high light (HL) [65], light quality change (PSI-II vs PSI and PSII-I vs PSII) [71], UV light (UV) [74], low nitrogen (LN) [104], sulphur limitation (S-) [65]and potassium limitation (K-) [116] stresses. The data set used for the analysis is found in Supplementary data, Table S1. The selected metabolites are also presented in Fig. 1.

## Slide 2
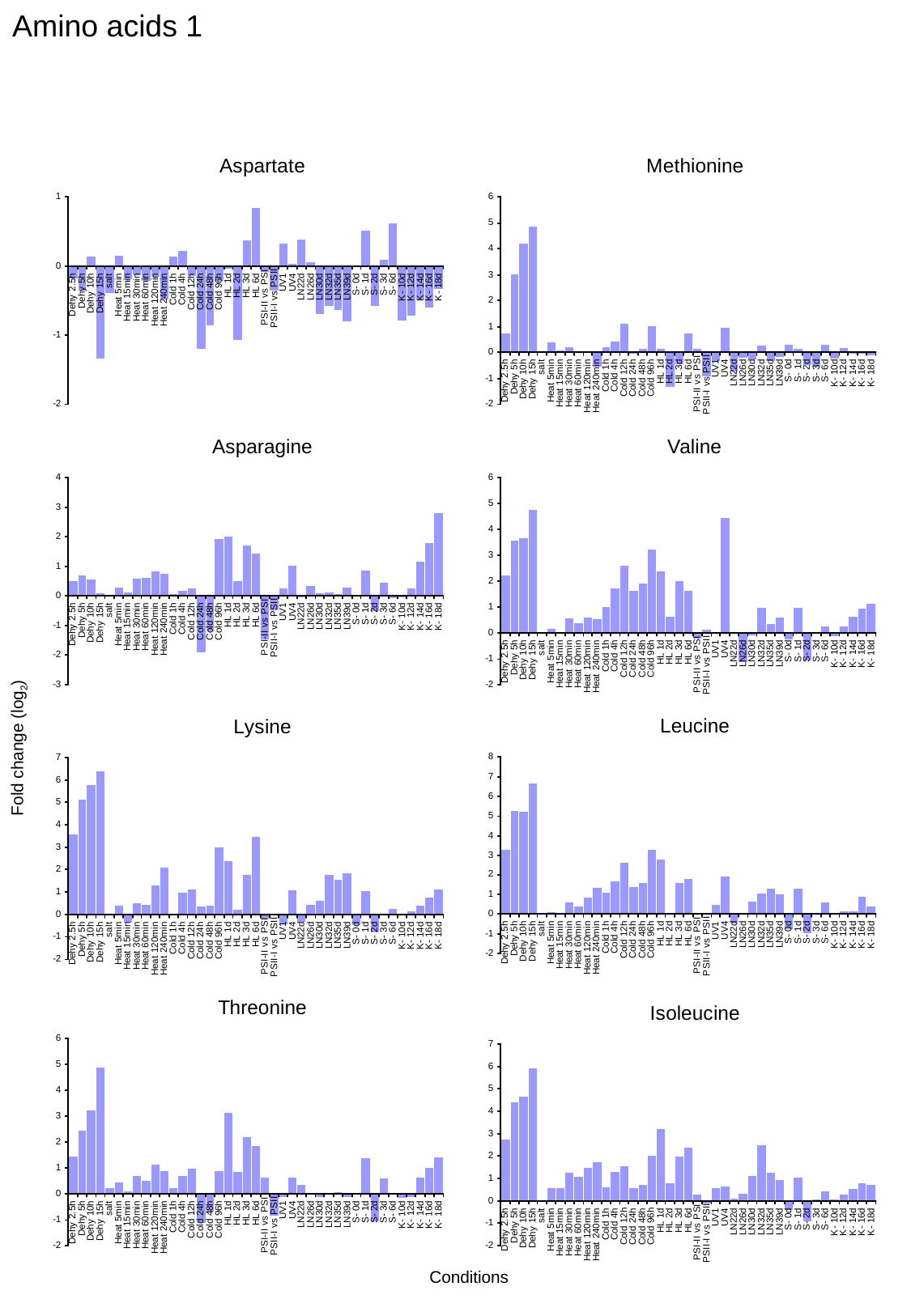

Amino acids 1
Fold change (log2)
Conditions

## Slide 3
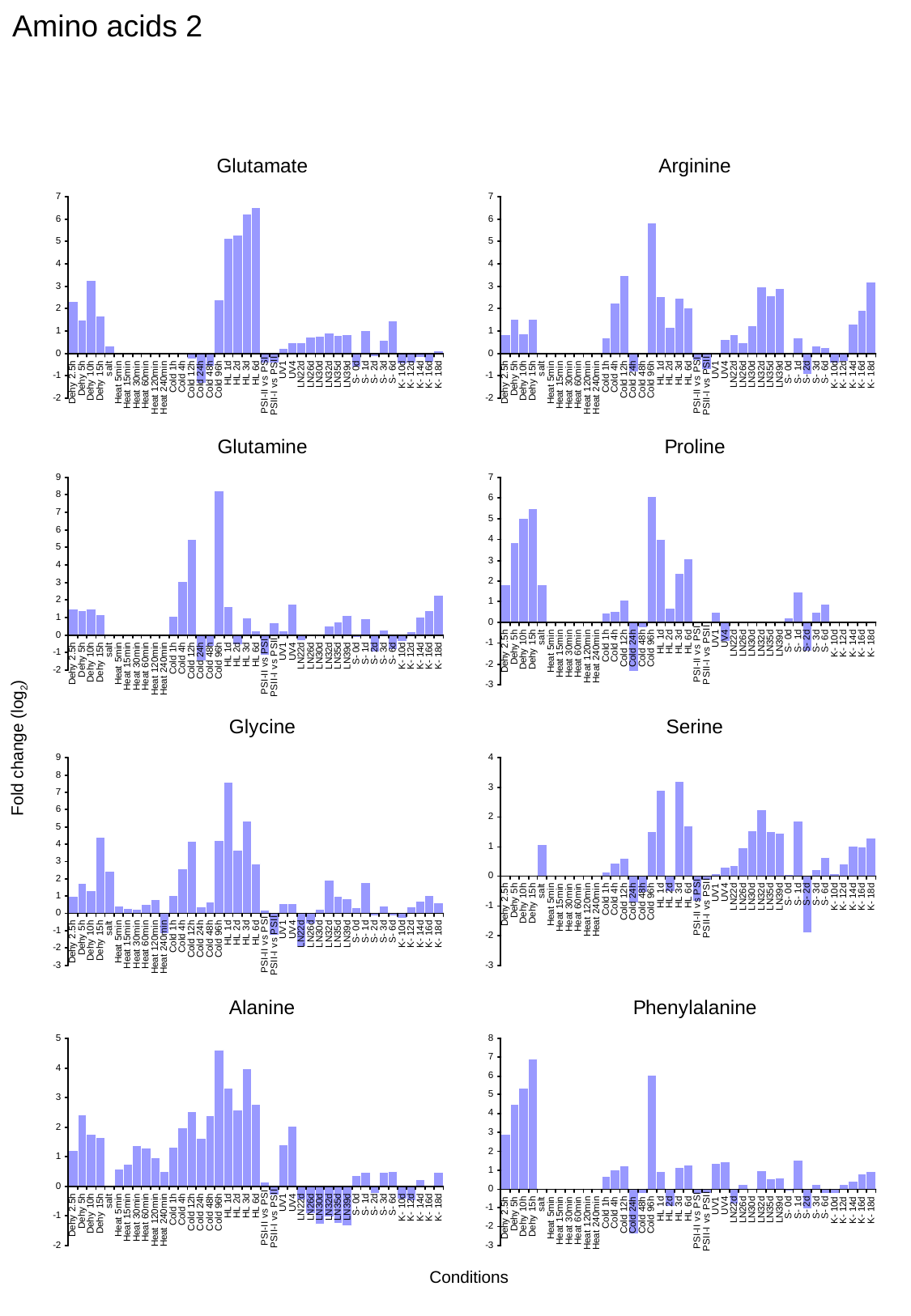

Amino acids 2
Fold change (log2)
Conditions

## Slide 4
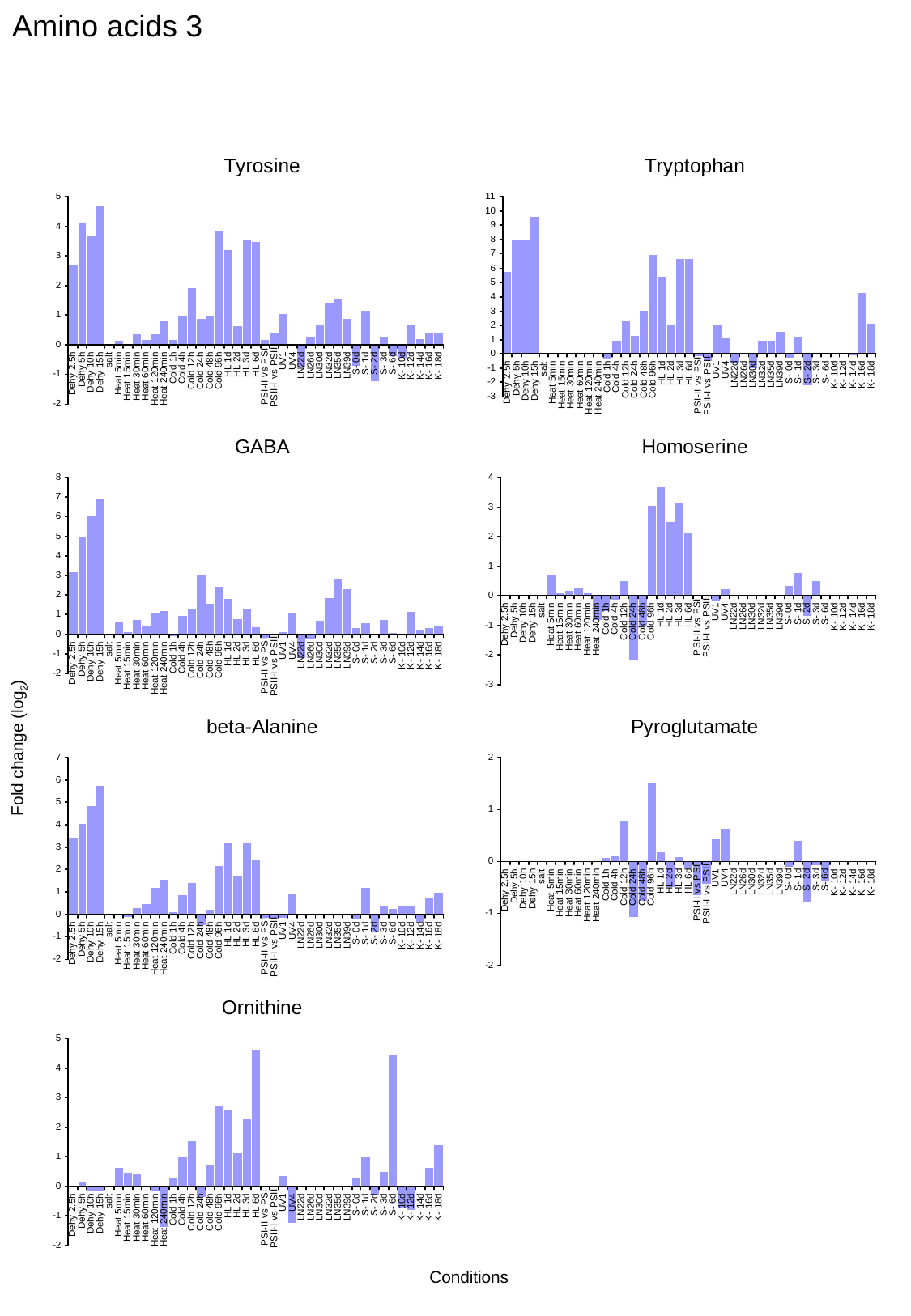

Amino acids 3
Fold change (log2)
Conditions

## Slide 5
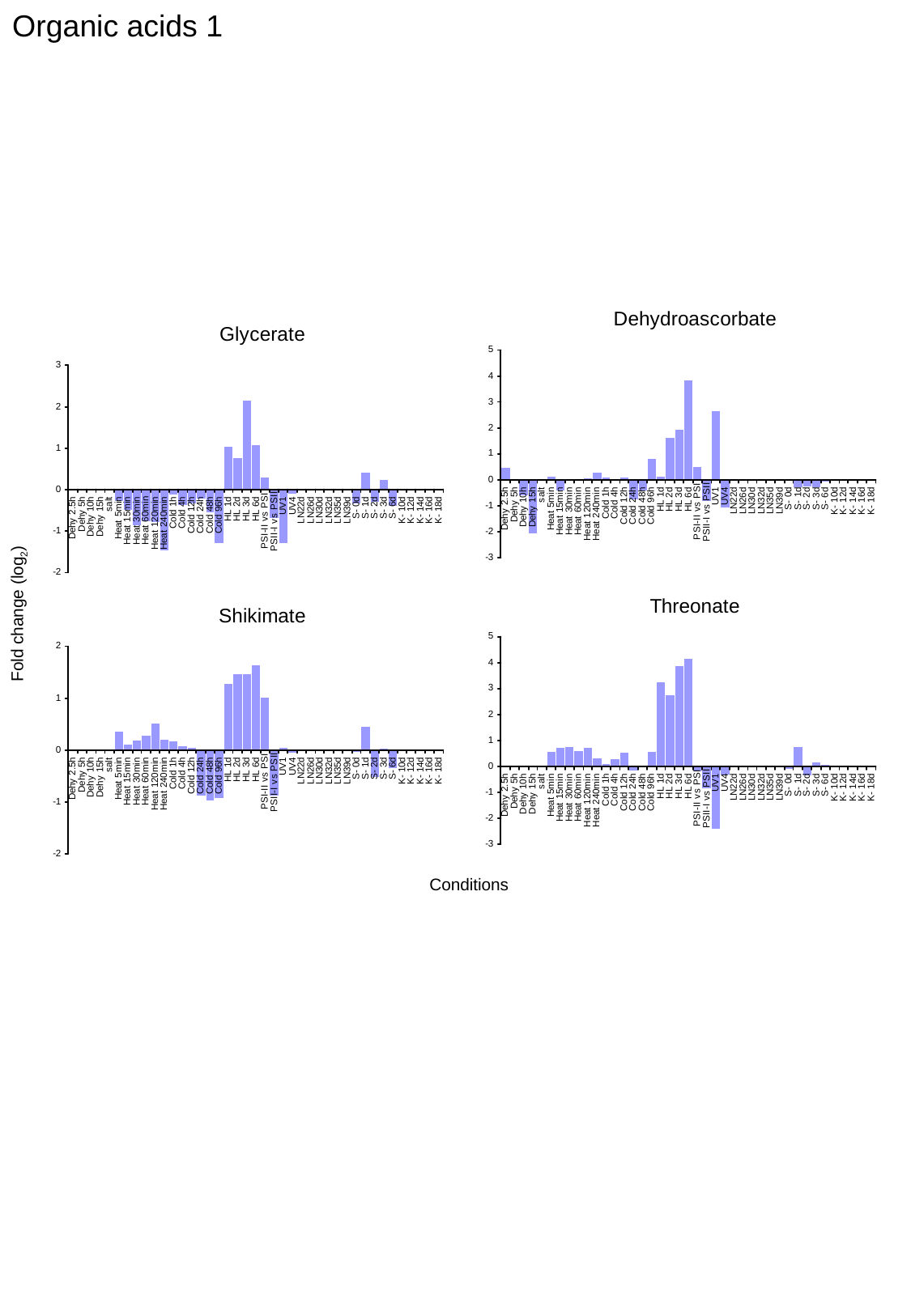

Organic acids 1
Fold change (log2)
Conditions

## Slide 6
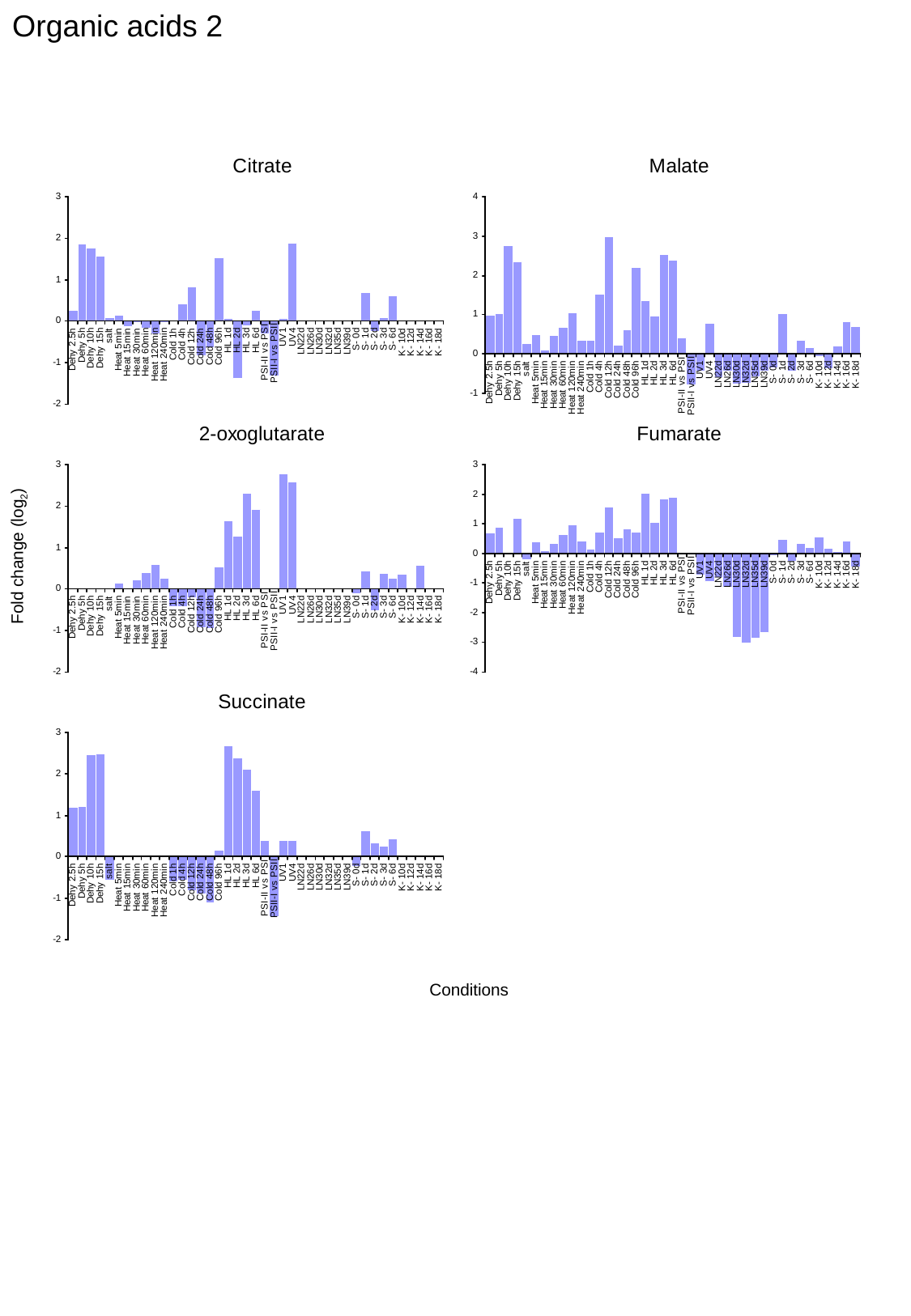

Organic acids 2
Fold change (log2)
Conditions

## Slide 7
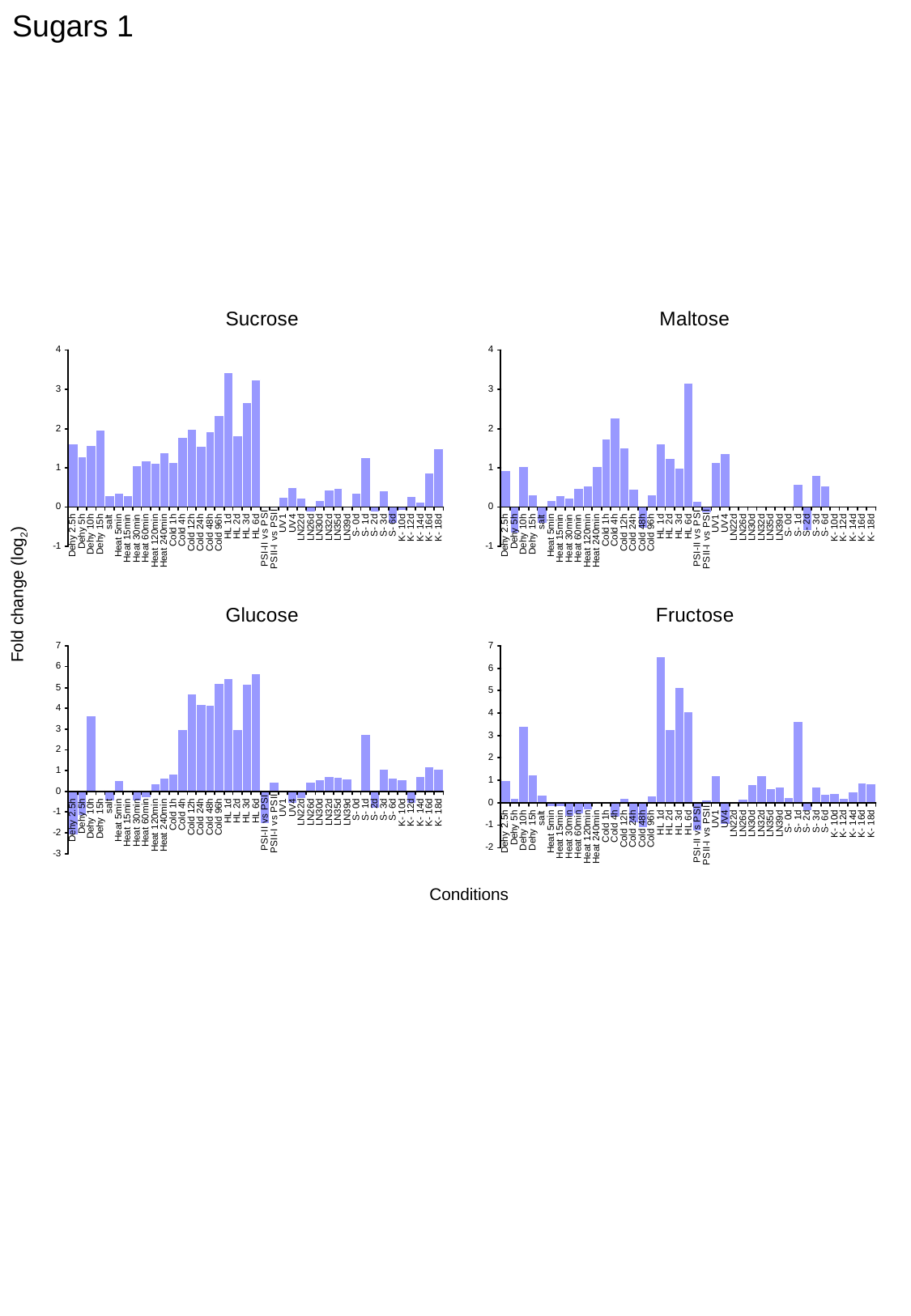

Sugars 1
Fold change (log2)
Conditions

## Slide 8
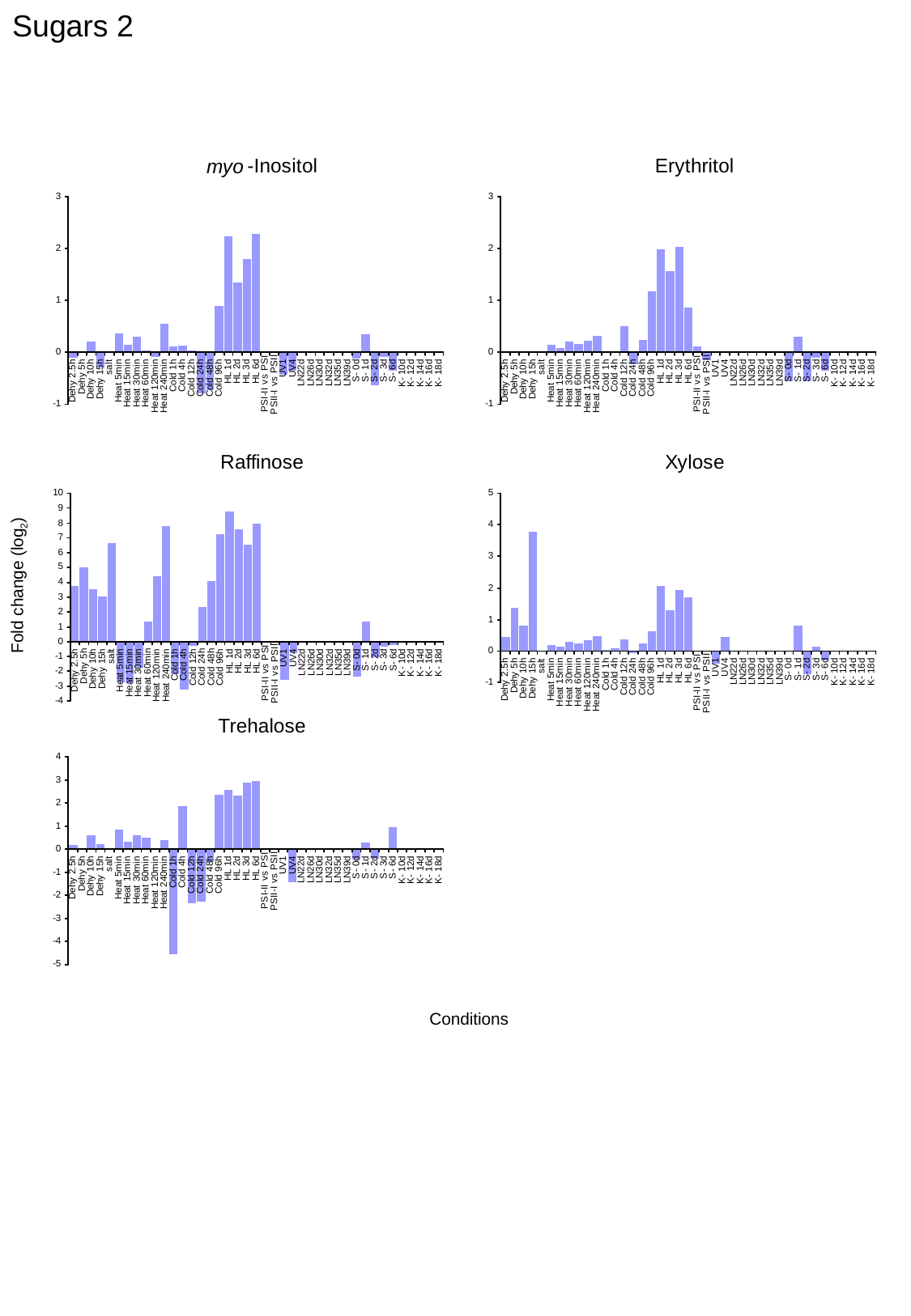

Sugars 2
Fold change (log2)
Conditions

## Slide 9
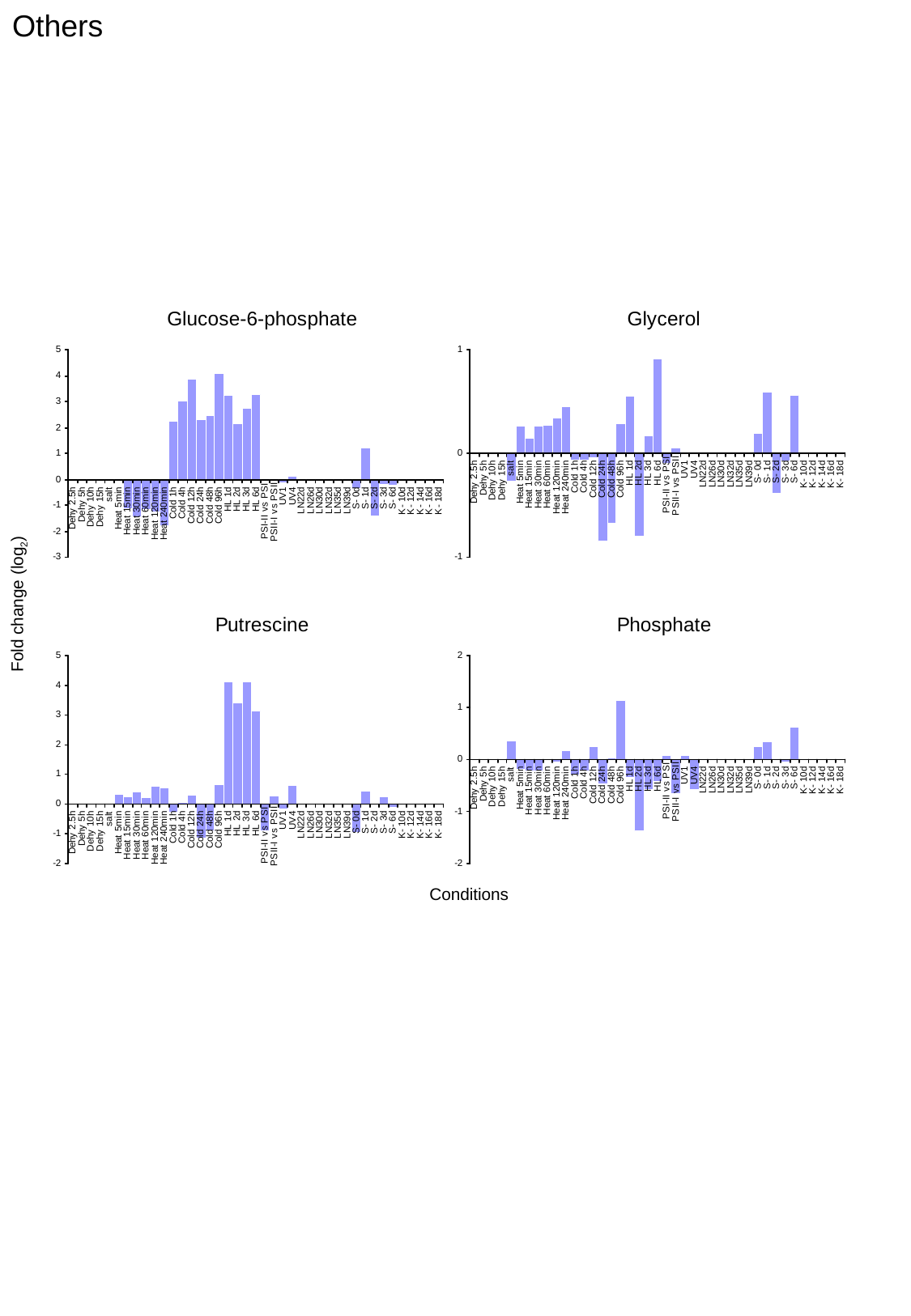

Others
Fold change (log2)
Conditions
